# Risk Factors for Falls in Elderly Patients with Moderate-to-Severe Dementia.

**DOI:** 10.1093/geroni/igaf122.3841

**Published:** 2025-12-31

**Authors:** Huixian Li, Tianyi Luo, Yanyan Wang

**Affiliations:** West ChinaHospital, Sichuan University, Chengdu, Sichuan, China (People’s Republic); West ChinaHospital, Sichuan University, Chengdu, Sichuan, China (People’s Republic); West ChinaHospital, Sichuan University, Chengdu, Sichuan, China (People’s Republic)

## Abstract

Dementia is a progressive neurodegenerative disease that affects millions of older adults worldwide, leading to cognitive and functional decline and significantly increases the risk of falls. While physiological risk factors are well documented, evidence on environmental and behavioral contributors remains limited. Using data from the Chengdu Long-term Care Insurance (LTCI) database (2018–2022), we conducted a cross-sectional study to evaluate factors influencing fall risk among older adults with moderate-to-severe dementia. We applied univariate and multivariate logistic regression analyses to examine the associations of residential environment, physical function, and neuropsychiatric symptoms with fall risk. A total of 5,806 participants were included, with a median age of 82.0 years (IQR, 76.0–87.0); 62.2% were female. Multivariate logistic regression analyses showed that participants living at home, compared with those in skilled nursing facilities, had a significantly higher risk of falls (adjusted odds ratio [aOR] = 2.15, 95% CI: 1.60–2.89). Other independent risk factors included aphasia (aOR = 2.11 [1.67–2.66]), dysphagia (aOR = 1.69 [1.32–2.17]), hallucinations/delusions (aOR = 1.30 [1.04–1.62]), genitourinary disorders (aOR = 1.36 [1.08–1.71]), and rheumatic autoimmune diseases (aOR = 1.31 [1.02–1.68]). Notably, participants with mental illness had a lower risk of falls (aOR = 0.53 [0.43–0.66]), which may reflect more intensive medical care and management in this population. Our findings indicate that fall risk assessment and prevention for individuals with moderate-to-severe dementia should systematically incorporate residential environment, neuropsychiatric symptoms, and functional impairments. Addressing these factors is essential for improving patient safety, ensuring high-quality medical services, and reducing caregiver burden.

